# CXC Chemokines as Therapeutic Targets and Prognostic Biomarkers in Skin Cutaneous Melanoma Microenvironment

**DOI:** 10.3389/fonc.2021.619003

**Published:** 2021-03-09

**Authors:** Xuezhi Zhou, Manjuan Peng, Ye He, Jingjie Peng, Xuan Zhang, Chao Wang, Xiaobo Xia, Weitao Song

**Affiliations:** Hunan Key Laboratory of Ophthalmology, Eye Center of Xiangya Hospital, Central South University, Changsha, China

**Keywords:** CXC chemokine, therapeutic targets, prognostic biomarkers, skin cutaneous melanoma, microenvironment

## Abstract

**Background:**

Skin Cutaneous Melanoma (SKCM) is a tumor of the epidermal melanocytes induced by gene activation or mutation. It is the result of the interaction between genetic, constitutional, and environmental factors. SKCM is highly aggressive and is the most threatening skin tumor. The incidence of the disease is increasing year by year, and it is the main cause of death in skin tumors around the world. CXC chemokines in the tumor microenvironment can regulate the transport of immune cells and the activity of tumor cells, thus playing an anti-tumor immunological role and affecting the prognosis of patients. However, the expression level of CXC chemokine in SKCM and its effect on prognosis are still unclear.

**Method:**

Oncomine, UALCAN, GEPIA, STRING, GeneMANIA, cBioPortal, TIMER, TRRUST, DAVID 6.8, and Metascape were applied in our research.

**Result:**

The transcription of CXCL1, CXCL5, CXCL8, CXCL9, CXCL10, and CXCL13 in SKCM tissues were significantly higher than those in normal tissues. The pathological stage of SKCM patients is closely related to the expression of CXCL4, CXCL9, CXCL10, CXCL11, CXCL12, and CXCL13. The prognosis of SKCM patients with low transcription levels of CXCL4, CXCL9, CXCL10, CXCL11, and CXCL13 is better. The differential expression of CXC chemokines is mainly associated with inflammatory response, immune response, and cytokine mediated signaling pathways. Our data indicate that the key transcription factors of CXC chemokines are RELA, NF-κB1 and SP1. The targets of CXC chemokines are mainly LCK, LYN, SYK, MAPK2, MAPK12, and ART. The relationship between CXC chemokine expression and immune cell infiltration in SKCM was closed.

**Conclusions:**

Our research provides a basis for screening SKCM biomarkers, predicting prognosis, and choosing immunotherapy.

## Introduction

Skin Cutaneous Melanoma is a common skin tumor caused by abnormal hyperproliferation of melanocytes (1). Its incidence varies with race, region, and age. The incidence of white people is much higher than that of black people, with the incidence of white people living in Queensland, Australia reaching 17/100,000 (2). In recent decades, the incidence of melanoma has continued to increase worldwide. It is the fastest-growing malignant disease in men and second only to lung cancer in women, with annual growth rates of 3–5% (3). Melanoma ranks 5th and 6th in malignant diseases in males and females, respectively, second only to adult leukemia in terms of risk of death (4). The median age of onset was 45–55 years old. Risk factors for melanoma include clear family history, melanoma history, multiple atypical or dysplastic nevi, and congenital genetic mutations (5). Sun exposure may also promote the development of melanoma (5). The association between molecular biological indicators and tumor prognosis has aroused great attention of researchers lately. Studies have revealed the relationship between the expression of S-100, Vimentin, and other proteins and tumor metastasis and prognosis (6). However, more effective therapeutic targets and more sensitive markers related to prognosis are still to be developed.

Chemokines are a superfamily composed of small molecules of cytokine-like proteins (7). CXC chemokines are an important member of this family (8). They occupy a major position in inflammation and damage repair and are closely linked with the occurrence, development, invasion, and metastasis of tumors (9). The function of CXC chemokines in tumors is deep association with the existence of ELR domains (10). CXC chemokines containing ELR domains are mainly concerned with the growth, proliferation, metastasis, and angiogenesis regulation of tumor cells. The diversity of functions of CXC chemokines without ELR domains in tumors may be related to the difference of ELR-CXC chemokine binding receptors. Therefore, we therefore speculate that CXC chemokines may also occupy and major position in various biological processes of SKCM.

In SKCM there are many updated reviews on molecular factors important for biology, drug targeting, and prognosis (BRAF, MEK, PD-1 et. al) (11–13). However, few studies have focused on the mechanism of CXC chemokine in SKCM and its potential value. Studies have reported the expression and function of some members of CXC chemokines in SKCM. However, so far it is still unknown which CXC can be used as a biomarker for prognosis and a target for immunotherapy for SKCM. With the continuous update and iteration of a variety of biological detection methods, it is possible to comprehensively analyze CXC chemokines.

Hence, the focus of this research is to find biological targets from CXC that can be used as the diagnosis and treatment of SKCM. In addition, we hope to discover the specific molecular mechanism of CXC affecting prognosis of SKCM through bioinformatics analysis.

## Materials and Methods

### ONCOMINE

ONCOMINE (http://www.oncomine.org) is a great tumor gene chip database, which contains multiple functions include finding outliers, predicting co-expressed genes, and analyzing gene expression differences (14). It can also be classified according to clinical information such as tumor stage, grade, and tissue type, and can also be used to search for possible diagnostic biomarkers and therapeutic targets. In this study, figure was exported to show the expression difference of CXC chemokines in SKCM through analysis from this website. The expression differences of CXC chemokines in SKCM were analyzed by a Student t-test. ONCOMINE contains 715 datasets and 86,733 samples.

### UALCAN

UALCAN (http://ualcan.path.uab.edu/index.html) can effectively analyze and mine cancer data on-line (15). The tumor-related data of the TCGA database is the basis of data mining on this website. The function of UALCAN includes biomarker identification, expression profile analysis, survival analysis, *etc*. In our study, the SKCM data set was import into UALCAN Expression Analysis module to evaluate the CXC chemokines expression. Student t-test was used to analyzed SKCM data.

### GEPIA

GERIA (http://gepia.cancer-pku.cn/index.html) is a newly developed interactive web server for analyzing the RNA sequencing expression data of 9,736 tumors and 8,587 normal samples from the TCGA and the GTEx projects, using a standard processing pipeline (16). It fills the gap of big data information in cancer genomics. GEPIA analyzed RNA sequencing expression data from 9,736 tumors and 8,587 normal samples from the TCGA and GTEx projects. The expression data of TCGA and GTEx are calculated under the same pipeline, which can be directly analyzed in a very comprehensive way. The database is an open public database. In this study, we used GEPIA’s “Single Gene Analysis” to analyze the differential expression of CXC chemokines in tumor tissues and normal tissues, as well as pathological stage Analysis and prognosis Analysis. “Multiple Gene Comparison” was used for the polygenic comparative analysis of CXC chemokines in the “SKCM” data set. Student t-test was used to explore SKCM data.

### STRING

STRING (https://string-db.org/) is a database that searches for known interactions between proteins and predicts their interactions (17). The database, available for 2,031 species, contains 9.6 million proteins and 13.8 million protein–protein interactions. It contains experimental data, PubMed abstracts, and other database data, as well as results predicted by bioinformatics methods. In this study, STRING was used for PPI analysis of CXC chemokines and peripheral interaction gene.

### GeneMANIA

The function of GeneMANIA (http://www.genemania.org) is to predict the interactions between proteins, including Predicted, Physical Interactions, co-location, co-expression, Pathway, Shared protein domains, and Genetic Interactions (18). GeneMANIA has almost 2,277 networks that collectively contain nearly 600 million interactions covering almost 163,599 genes.

### cBioPortal

cBioPortal (www.cbioportal.org) integrates data from 126 tumor genome studies (19). These included large tumor research projects such as TCGA and ICGC, which included data from 28,000 samples, and some samples with phenotypic information such as clinical prognosis. Genetic variation, gene network, and co-expression of CXC chemokines in SKCM were analyzed by cBioPortal.

### TIMER

TIMER (https://cistrome.shinyapps.io/timer/) using a deconvolution algorithm from the gene expression profile (TIICs) concluded that tumor-infiltrating immune cells in abundance (20). Gene expression data of 10,897 TCGA samples from 32 cancer types were reanalyzed to estimate the abundance of six TIIC subsets (B cells, CD4+T cells, CD8+T cells, macrophages, neutrophils, and dendritic cells) to establish a link between tumor immunity and genomic data. The web server provides the abundance of immune infiltrates estimated by multiple immune-deconvolution methods and allows users to dynamically generate high-quality graphics to fully explore the immunological, clinical, and genomic characteristics of tumors.

### TRRUST

TRRUST (https://www.grnpedia.org/trrust/) is a record of transcription factors regulating the relationship database (21). TRRUST contains 8,444 and 6,552 TF-target regulatory relationships of 800 human TFs and 828 mouse TFs (21). It includes not only the corresponding target genes of transcription factors but also the regulatory relationship between transcription factors. At present, the database only stores regulatory information related to humans and mice, and these regulatory relations are sorted out from the literature through text mining.

### DAVID 6.8

DAVID 6.8 integrates biological data and analysis tools to provide systematic and comprehensive biological functional annotation information for the large-scale gene or protein lists (hundreds of gene or protein ID lists) to help users extract biological information from DAVID 6.8 (https://david.ncifcrf.gov/summary.jsp) (22). GO enrichment analysis and KEGG analysis on CXC chemokines and 50 closely related genes were performed by applying DAVID.

### Metascape

Metascape (http://metascape.org) integrates more than 40 bioinformatics databases (23). It not only includes bio-pathway enrichment analysis, protein interaction network structure analysis, and abundant gene annotation functions but also presents the results in high-quality graphical language that biologists can easily understand. The enrichment analysis of CXC chemokines and highly interacting genes were verified by the Express Analysis module.

### qRT-PCR Analysis

10 SKCM tissues and 12 normal tissues were enrolled in our research to verify the CXC chemokines expression. We obtained consent of patients and ethical approval from the Xiangya hospital ethics committee. All procedures were executed by the ethics guidelines and regulations. We extracted total RNA of the tissue by using Trizol reagent (Invitrogen, CA, USA). qRT-PCR was performed with SYBR^®^ Green dye (TaKaRa, Shiga, Japan). The primer sequences of relative genes are provided in **Supplementary Table 1**.

## Results

### CXC Chemokines in SKCM Patients

**Figure 1:**
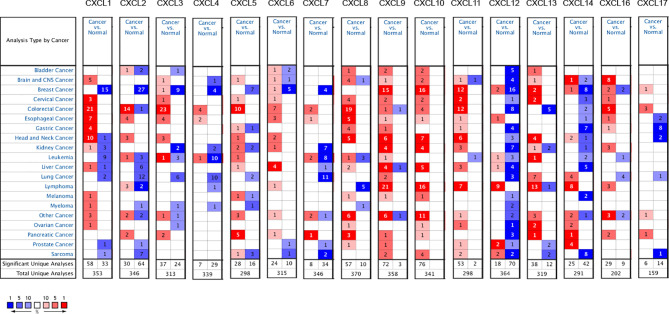
mRNA levels of CXC chemokines in SKCM (ONCOMINE). The panel shows the numbers of datasets with statistically significant mRNA over-expression (red) or downregulated expression (blue) of CXC chemokines.

The expression differences of 16 CXC chemokines between SKCM patients and controls were analyzed in the Oncomine database (**Figure 1** and **Table 1**). Analysis results showed that the transcription levels of CXCL1, CXCL5, CXCL8, CXCL9, CXCL10, and CXCL13 in SKCM patients were significantly increased compared with normal skin tissues. Risker et al. confirmed that the transcription level of CXCL1, CXCL5, CXCL8, and CXCL13 in SKCM patients were significantly higher than those in normal skin tissues (24). Haqq et al. also proved that the transcription of CXCL1, CXCL9, and CXCL10 in melanoma patients is significantly higher than that in normal skin tissues with a P-value of 0.005, 6.75E-4, and 2.52E-4 (25). Talatov et al. also confirmed that the transcription level of CXCL8 was significantly increased with a P-value of 0.004 (26). There was no significant difference in the transcriptional levels of SKCM patients and normal people among CXCL2, CXCL3, CXCL4, CXCL6, CXCL7, CXCL11, CXCL12, CXCL14, CXCL16, and CXCL17.

**Table 1:** The mRNA levels of CXC in SKCM and normal skin tissues at transcriptome level.

TLR	Type	Fold change	*P*-value	*t*-test	References
CXCL1	SKCM Melanoma	4.096 12.232	0.003 0.004	3.535 4.100	(21) (22)
CXCL5	SKCM	6.945	1.73E-4	4.528	(21)
CXCL8	SKCM SKCM	5.651 2.559	0.004 0.004	3.239 2.837	(21) (23)
CXCL9	SKCM	5.144	6.75E-4	6.321	(22)
CXCL10	SKCM	5.651	2.52E-4	6.311	(22)
CXCL13	SKCM	11.238	0.037	2.166	(21)

**Figure 2:**
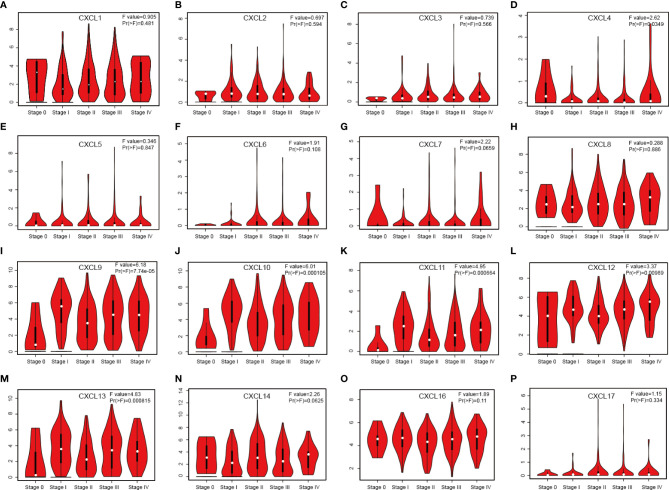
Correlation between different expressed CXC chemokines and the pathological stage of SKCM patients **(A–P)** (GEPIA). *P* value less than 0.05 indicate significant difference.

Relationship between 16 CXC chemokines and the pathological stage of SKCM was assessed by GEPIA. The results showed that CXCL4 (P = 0.0349), CXC9 (P = 7.74E-05), CXC10 (P = 0.000105), CXC11 (P = 0.000664), CXC12 (P =0.00989), and CXC13 (P =0.000815) were notably associated with the pathological stage of SKCM (**Figure 2**). High expression of CXCL4, CXCL9, CXCL10, CXCL11, CXCL12, and CXCL13 promotes the progress of SKCM. It concluded that CXC chemokines are extremely closely connected with the pathological progress of SKCM.

### CXC Chemokines in Primary SKCM and Metastasis SKCM Patients

**Figure 3:**
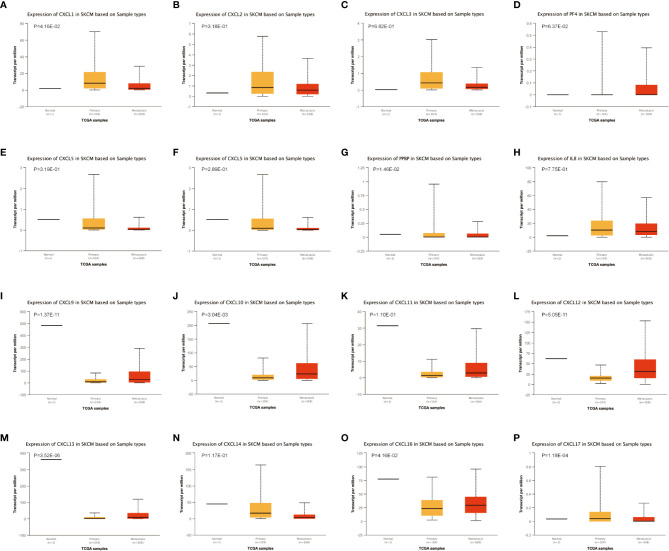
The transcription of CXC chemokines in SKCM (UALCAN). The transcriptional levels of **(A)** CXCL1 and **(G)** CXCL7 in primary SKCM tissues were significantly elevated compared with metastasis SKCM patients while the transcriptional levels of **(I)** CXCL 9, **(J)** CXCL 10, **(L)** CXCL 12, **(M)** CXCL 13, and **(O)** CXCL 16 were significantly reduced. The p value was set at 0.05. There were no differences in transcriptional levels of **(B)** CXCL 2, **(C)** CXCL 3, **(D)** PF4, **(E)** CXCL 5, **(F)** CXCL6, **(H)** IL 8, **(K)** CXCL11, **(N)** CXCL 14, and **(P)** CXCL 17 between primary SKCM and metastasis SKCM patients. The p value was set at 0.05.

**Figure 4:**
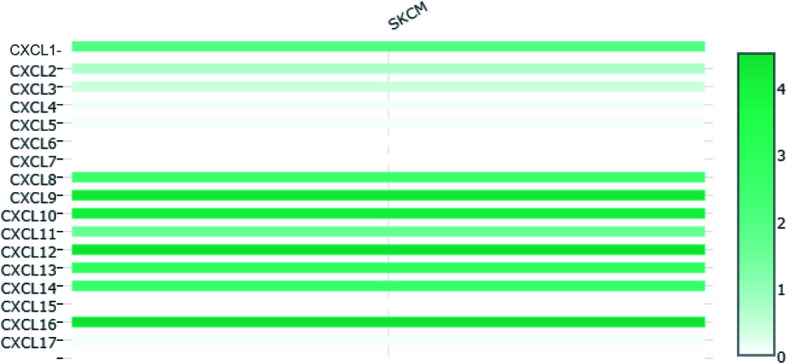
The relative level of CXC chemokines in SKCM.

The difference in CXC chemokine expression levels between primary SKCM and metastatic SKCM patients was analyzed by UALCAN (**Figure 3**). Analysis results indicated that the CXCL1 (*P* = 4.16E-02) and CXCL7 (*P* = 1.46E-02) level in primary SKCM patients was remarkably higher than metastasis SKCM patients (*P* = 4.16E-02). However, the expression level of CXCL9 (*P* = 1.37E-11), CXCL10 (*P* = 3.04E-03), CXCL12 (*P* = 5.05E-11), CXCL13 (*P* = 3.52E-06), and CXCL16 (*P* =1.18E-04) in patients with metastasis SKCM is notably higher than that of patients with primary SKCM. We concluded that CXCL1, CXCL 7, CXCL 9, CXCL 10, CXCL 12, CXCL 13, and CXCL 16 have obvious differences in expression in patients with primary SKCM and metastasis SKCM. No obvious difference has been seen in the expression levels of CXCL2, CXCL3, CXCL4, CXCL5, CXCL6, CXCL8, CXCL11, CXCL14, and CXCL17 in patients with primary SKCM and metastasis SKCM. We also analyzed the expression levels of each gene of the CXC chemokine family in SKCM tissue through the GEPIA website (**Figure 4**). The analysis results revealed that CXCL12 and CXCL16 level in SKCM patients were highest.

### CXC Chemokines Affect Prognostic of SKCM Patients

**Figure 5:**
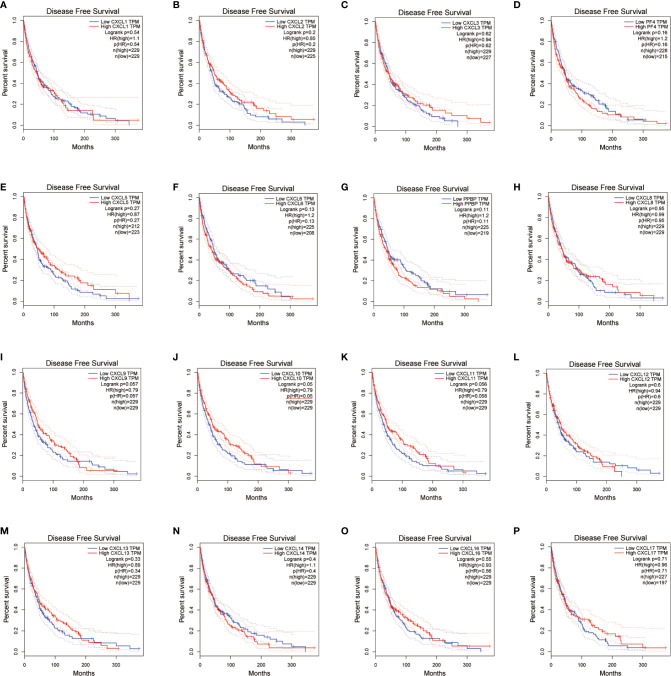
The prognostic value of different expressed CXC chemokines in SKCM patients in the disease free survival curve **(A–P)** (GEPIA).

**Figure 6:**
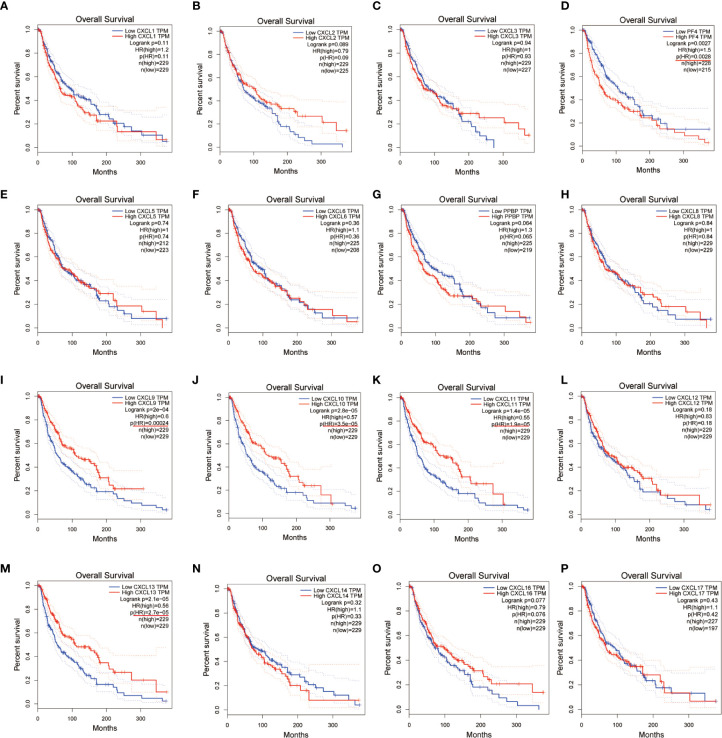
The prognostic value of CXC chemokines in SKCM patients in the overall survival curve **(A–P)** (GEPIA).

We evaluated the worth of different CXC chemokines in SKCM clinical outcomes by using GEPIA. No obvious correlation between the level of CXC chemokine family transcription and disease-free survival time in SKCM patients was found (**Figure 5**). The worth of CXC chemokines with different expressions in the total survival of SKCM patients was assessed (**Figure 6**). The results showed that low expression of CXCL4 (*p* = 0.0028), CXCL9 (*P* = 0.00024), CXCL10 (*P* = 2.8E-05), CXCL11 (*P* =1.9e-05), and CXCL13 (*P* = 2.7e-05) was remarkably related to longer overall survival in SKCM.

### Gene Changes, Adjacent Gene Networks, and Interaction of CXC Chemokines in SKCM

**Figure 7:**
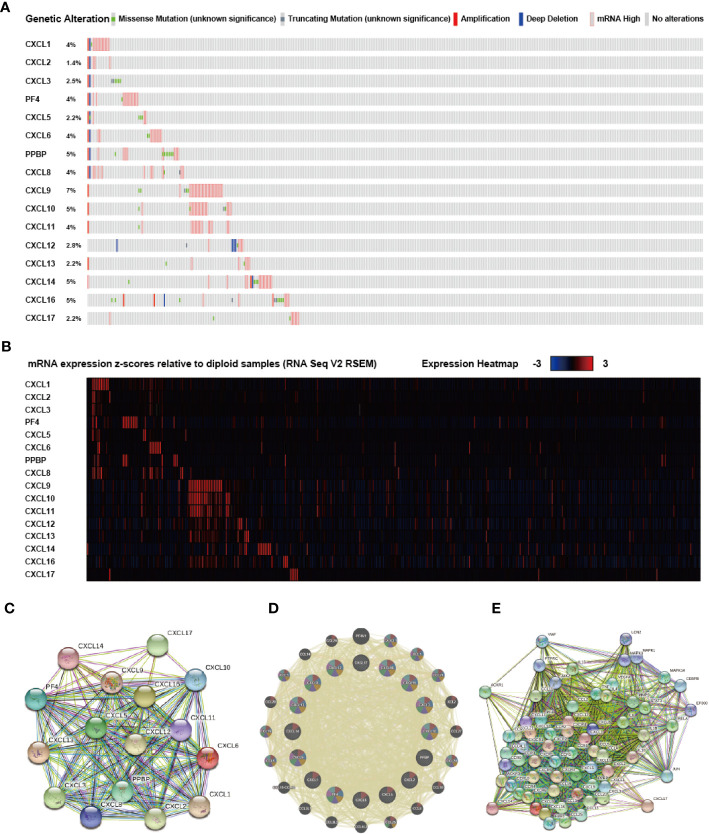
Genetic alteration, mRNA expression, and interaction analyses of different expressed CXC chemokines in SKCM patients. **(A)** Summary of alterations in different expressed CXC chemokines in SKCM. **(B)** mRNA expression heat map of different expressed CXC chemokines in SKCM. **(C, D)** Protein-protein interaction network of different expressed CXC chemokines. **(E)** Gene-gene interaction network of different expressed CXC chemokines and 50 most frequently altered neighboring genes.

An exhaustive molecular characterization excavation of CXC chemokines differentially expressed was performed. The genetic changes of differentially expressed CXC chemokines were analyzed applying the TCGA data set. The analytical results showed that CXCL1, CXCL2, CXCL3, CXCL4, CXCL5, CXCL6, CXCL7, CXCL8, CXCL9, CXCL10, CXCL11, CXCL12, CXCL13, CXCL14, CXCL16, CXCL17 were changed in 4, 1.4, 2.5, 4, 2.2, 4, 5, 4, 7, 5, 4, 2.8, 2.2, 5,5, and 2.2% of the queried SKCM samples, respectively (**Figures 7A, B** **)**. The most common change in these samples was increased mRNA expression. Also, PPI network analysis was performed for CXC chemokines and Strings with different expressions to explore their possible interactions. Several 16 nodes and several 11 edges were acquired from the PPI network (**Figure 7C**). PPI analysis results showed that the PPI enrichment *P*-value was <1.0e-16. GeneMANIA analysis showed that the effect of 16 CXC chemokines was mainly associated with chemokine receptor binding and activity (**Figure 7D**). Besides, String analyzed the top 50 most regularly interacting neighboring genes related to 16 CXC chemokines. The results showed that CXCR4, CXCR2, CCR5, IL10, CXCR1, CXCR5, CCL5, ACKR3, CCL19, CXCR6, IL4, IL1B, CCL21, CCL11, CCL11, CCL20, CCL25, RELA, CCL1, IL6, IL13, STAT3, MMP9, CCR4, CCR3, ACKR1, CCL2, CCR5, CCL4L1, CCR2, MAPK14, TNF, VEGFA, CCR7, CC R1, FPR2, JUN, CX3CL1, CEBPB, CCR10, VWF, PTPRC, MAPK1, JAK2, CX3CR1, MAPK3, CCR9, LCN2, and EP300 are mainly related to the regulation and capability of CXC chemokines in SKCM patients (**Figure 7E**).

### Biological Process Analysis of CXC Chemokines

**Figure 8:**
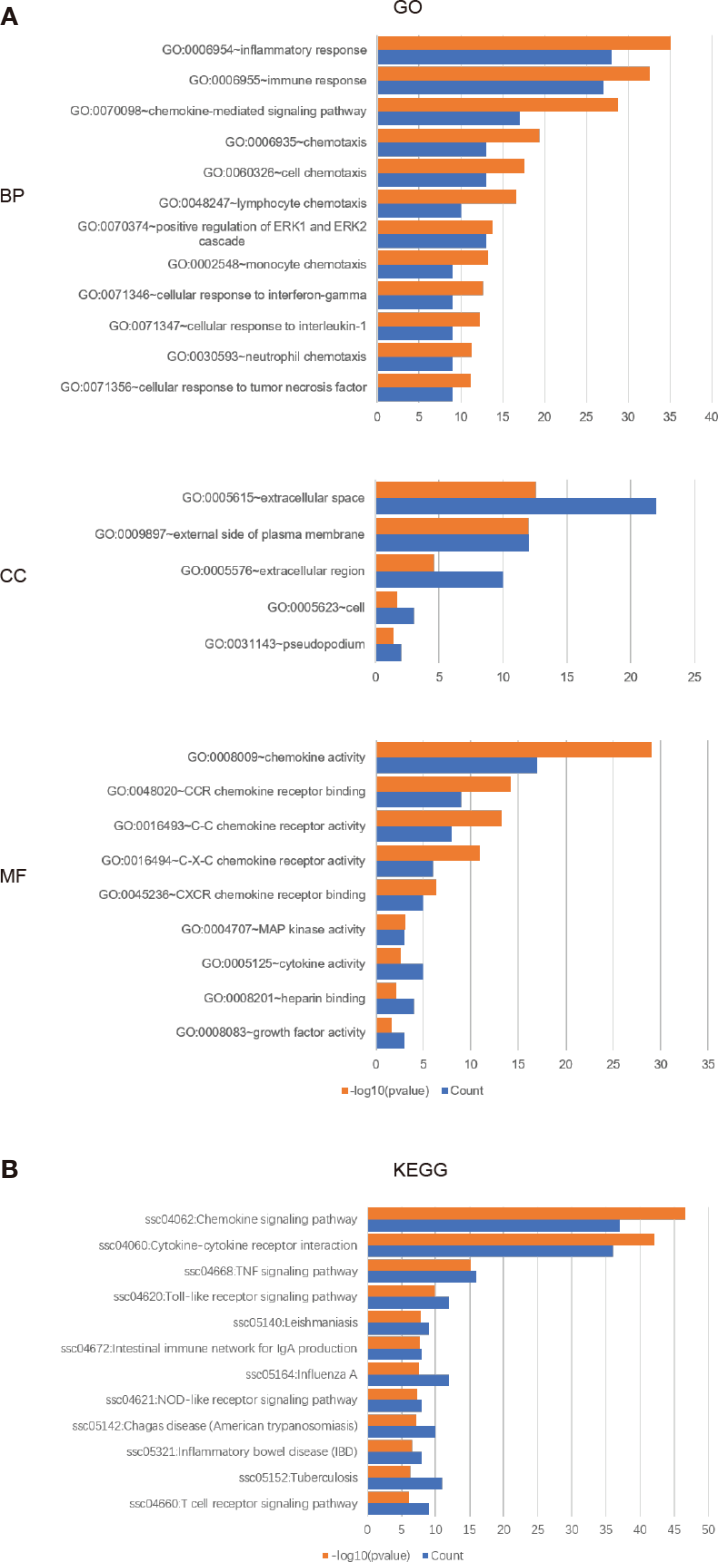
The enrichment analysis of different expressed CXC chemokines and 50 most frequently altered neighboring genes in SKCM (David 6.8). **(A)** Bar plot of GO enrichment in biological process terms, cellular component terms, and molecular function terms. **(B)** Bar plot of KEGG enriched terms.

The enrichment analysis of CXC chemokines and its closely interacting neighboring genes is performed by DAVID 6.8 and Metascape. **Figure 8A** shows the first 10 items using DAVID 6.8 for the functional enrichment analysis. In the BP category, inflammatory response, immune response, cytokine-mediated signaling pathway, leukocyte chemotaxis, positive regulation of ERK1 and ERK2, and cellular response to interferon-*γ*, interleukin-1, and tumor necrosis factor were associated with SKCM oncogenesis and progression. In the CC category, the extracellular space, external side of the plasma membrane, extracellular region, cell, and Pseudopodium are the five functional enrichment projects. In the MF category, the CXC chemokines and its adjacent genes are mostly enriched in CXC chemokine receptor binding, C-C chemokine receptor activity, C-X-C chemokine receptor activity, CXCR Chemokine receptor binding, MAP kinase activity, cytokine activity, heparin-binding, and growth factor activity. KEGG pathway analysis was also performed, and the results showed the chemokine signaling pathway, cytokine receptor interaction, TNF signaling pathway, toll-like receptor signaling pathway, Leishmaniasis, Intestinal Immune Network for IgA production, Influenza, and NOD-like receptor signaling Pathway, Chagas disease, inflammatory disease, tuberculosis, T cell signaling pathway are closely related to tumor formation (**Figure 8B**).

**Figure 9:**
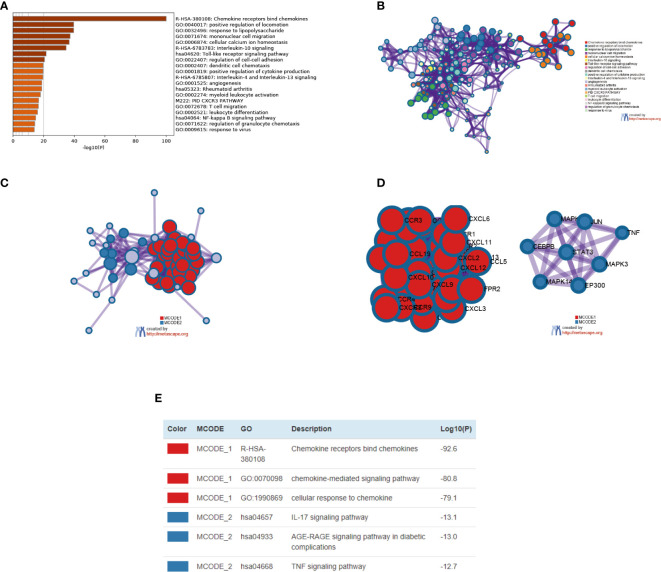
The enrichment analysis of different expressed CXC chemokines and 50 most frequently altered neighboring genes in SKCM patients (Metascape). **(A)** Bar graph of top 20 enriched terms across different expressed CXC chemokines and 50 most frequently altered neighboring genes, colored by p-values. **(B)** Network of enriched terms, colored by cluster ID. **(C–E)** Protein–protein interaction network and MCODE components identified in different expressed CXC chemokines and 50 most frequently altered neighboring genes.

We also used Metascape for enrichment analysis. The functions of CXC chemokines and their adjacent genes are mostly focused on chemokines bind chemokines, positive regulation of locomotion, response to lipopolysaccharide, mononuclear cell migration, cellular calcium ion homeostasis, Interleukin-10 signaling, Toll-like receptor signaling pathway, *etc.* (**Figures 9A, B** **)**. In order to explore the interaction between 16 CXC chemokines and its closely related gene, we analyzed the related data using the PPI network and mCODE components (**Figures 9C, D** **)**. The result showed that the CXC chemokines biological functions are mainly associated with chemokine receptors bind chemokines, chemokine-mediated signaling pathways, cellular response to chemokine, IL-7 signaling pathway, AGE-RAGE signaling pathway, and TNF signaling pathway (**Figure 9E**).

### Transcription Factor Targets and Kinase Target Analysis

TRRUST and LinkedOmics databases were used to explore possible transcription factors and kinase targets for differential expression of CXC chemokines. TRRUST contains CXCL 1, CXCL 2, CXCL 5, CXCL 8, CXCL 10, CXCL 12, and CXCL 14. Three transcription factors (RELA, NF-KB1, and SP1) were found to significantly regulated CXC chemokines in SKCM (**Table 2**). The transcription factors RELA and NFKB1were identified as key regulators of CXCL1, CXCL2, CXCL5, CXCL 8, CXCL10, and CXCL12. One of the main transcription factors regulating CXCL1, CXCL5, and CXCL 14 transcription in SKCM is SP1. LinkedOmics was utilized to analyze the kinase target of CXC chemokines (**Table 3**). ROCK1 and RPS6KB1 are among the most significant kinase targets for CXCL1. LCK and IKBKB are the most important kinase targets for CXCL2. MAPK12 and MYLK are the most important kinase targets for CXCL3. Both PAK1 and MAPK2 are key kinase targets of CXCL4. FER and JAK3 are most major kinase targets for CXCL5. BCR and MAPK14 are considered the key targets of the CXCL6 kinase target network. ADRBK2 and EIF2AK4 are considered as major targets of the CXCL8. LCK and SYK are the two largest targets in CXCL10, CXCL11, and CXCL9 kinase target network. LCK and LYN are mostly linked with CXCL12 and CXCL16. The CXCL13 kinase target network is mostly related to LCK and CSNK1E, while the CXCL14 kinase target network is mostly related to ART and PLK1. ART and NEK2 are considered as key targets of the CXCL17 kinase target network.

**Table 2:** Key regulated factor of CXC chemokines in SKCM (TRRUST).

Key TF	Description	Regulated gene	*P*-value	FDR
RELA	v-rel reticuloendotheliosis viral oncogene homolog A (avian)	CXCL1, CXCL2, CXCL5, CXCL8, CXCL10, CXCL12	1.09E-07	1.71E-07
NFKB1	nuclear factor of kappa light polypeptide gene enhancer in B-cells 1	CXCL1, CXCL2, CXCL5, CXCL8, CXCL10, CXCL12	1.14E-07	1.71E-07
SP1	Sp1 transcription factor	CXCL1, CXCL5, CXCL14	0.00683	0.00683

**Table 3:** The Kinase target networks of CXC chemokines in SKCM (LinkedOmics).

CXC chemokines	Enriched kinase target	Description	Leading EdgeNum	*P*-value
CXCL 1	Kinase_ROCK1 Kinase_RPS6KB1	Rho associated coiled-coil containing protein kinase 1 ribosomal protein S6 kinase B1	14 8	0 0
CXCL 2	Kinase_LCK Kinase_IKBKB	LCK proto-oncogene, Src family tyrosine kinase inhibitor of nuclear factor kappa B kinase subunit beta	26 8	0 0.0030030
CXCL 3	Kinase_MAPK12 Kinase_MYLK	mitogen-activated protein kinase 12 myosin light chain kinase	8 3	0.023569 0.015936
CXCL 4 (PF 4)	Kinase_PAK1 Kinase_MARK2	p21 (RAC1) activated kinase 1 microtubule affinity regulating kinase 2	19 6	0 0
CXCL 5	Kinase_FER Kinase_JAK3	FER tyrosine kinase Janus kinase 3	4 4	0.023256 0.034843
CXCL 6	Kinase_BCR Kinase_MAPK14	BCR, RhoGEF and GTPase activating protein mitogen-activated protein kinase 14	8 26	0.0031746 0
CXCL 8 (IL 8)	Kinase_ADRBK2 Kinase_EIF2AK4	G protein-coupled receptor kinase 3 eukaryotic translation initiation factor 2 alpha kinase 4	1 2	0.0042735 0.058366
CXCL 9	Kinase_LCK Kinase_SYK	LCK proto-oncogene, Src family tyrosine kinase spleen associated tyrosine kinase	24 17	0 0
CXCL 10	Kinase_LCK Kinase_SYK	LCK proto-oncogene, Src family tyrosine kinase spleen associated tyrosine kinase	22 15	0 0
CXCL 11	Kinase_LCK Kinase_SYK	LCK proto-oncogene, Src family tyrosine kinase spleen associated tyrosine kinase	20 16	0 0
CXCL 12	Kinase_LCK Kinase_LYN	LCK proto-oncogene, Src family tyrosine kinase LYN proto-oncogene, Src family tyrosine kinase	26 23	0 0
CXCL 13	Kinase_LCK Kinase_CSNK1E	LCK proto-oncogene, Src family tyrosine kinase casein kinase 1 epsilon	23 9	0 0
CXCL 14	Kinase_ATR Kinase_PLK1	ATR serine/threonine kinase polo like kinase 1	21 45	0 0
CXCL 16	Kinase_LCK Kinase_LYN	LCK proto-oncogene, Src family tyrosine kinase LYN proto-oncogene, Src family tyrosine kinase	21 23	0 0
CXCL 17	Kinase_ATR Kinase_NEK2	ATR serine/threonine kinase NIMA related kinase 2	27 5	0 0

### The Association of CXC Chemokines With Immune Cell Infiltration

**Figure 10:**
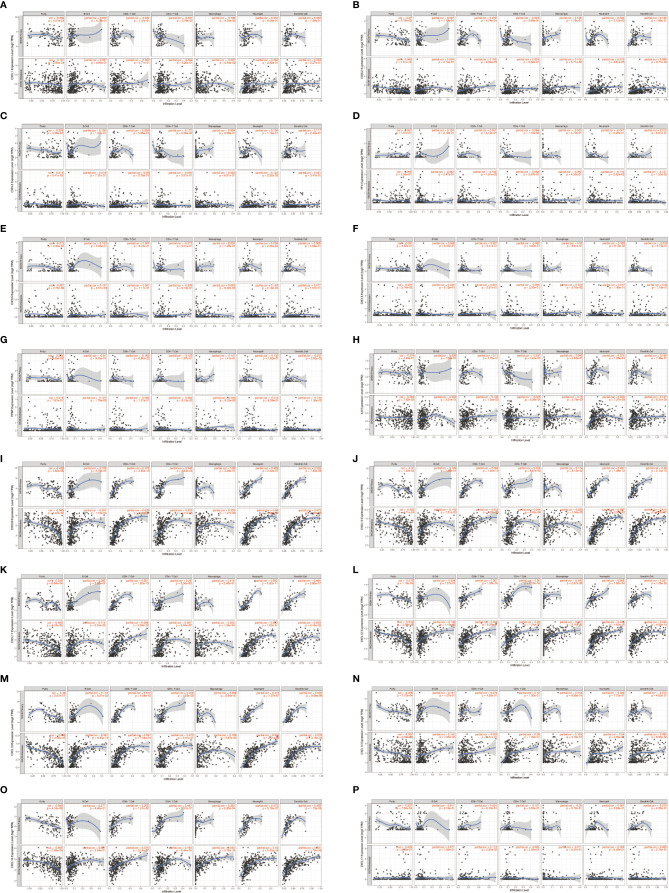
The correlation between different expressed CXC chemokines and immune cell infiltration in primary and metastasis SKCM patients **(A–P)** (TIMER).

Clinical outcome is related to inflammatory response and immune cell infiltration in SKCM patients, in which CXC chemokines are also involved. Timer database was applied to comprehensively quest whether CXC chemokines expression is associated with immune infiltration in SKCM patients (**Figrues 10A–P** and **Table 4**). The analysis results showed the correlation between CXCL1 expression and CD4+ T cell infiltration was negative in SKCM-primary patients (Cor = −0.207, *P* = 3.79E-02). The expression of CXCL2 was positively correlated with Neutrophil cell invasion in SKCM-primary patients (Cor = 0.293, *P* = 3.12E-03). CXCL2 is positively correlated with CD8+ T cells (Cor = 0.155, *P* = 4.32E-03) and Neutrophil cells (Cor = 0.215, *P* = 4.81e-05) infiltrates in SKCM-metastasis patients. CXCL3 is mostly related to Neutrophil cell invasion (Cor = 0.122, *P* = 2.22E-02) in SKCM-metastasis patients (**Figure 10C**). The correlation between the expression of CXCL4 and CD4+ T cell infiltration was negative in SKCM-primary patients (Cor = −0.266, *P* = 7.16E-03). In SKCM-metastasis, CXCL4 is negatively correlated with CD8+ T cells (Cor = −0.129, *P* = 1.81e-02) and Neutrophil cells (Cor = −0.119, *P* = 2.62e-02) and Dendritic cell (Cor = −0.131, *P* = 1.53e-02) infiltrates. CXCL5 was definitely related to Neutrophil cell infiltration in SKCM-primary patients (Cor = 0.228, *P* = 2.26E-02). In SKCM-metastasis, CXCL5 is positively correlated with invasion of B cells (Cor = 0.157, *P* = 3.51E-03) and Neutrophil cells (Cor = 0.163, *P* = 2.14e-03). The expression of CXCL7 in SKCM-primary was negatively correlated with Dendritic cells infiltration (Cor = −0.207, *P* = 3.82E-02). In SKCM-metastasis patients, CXCL7 is negatively correlated with B cells (Cor = −0.127, *P* = 1.79e-02) and Dendritic cells (Cor = −0.134, *P* = 1.30e-02) infiltrate. CXCL8’s expression was positively correlated with Neutrophil cell infiltration in SKCM-primary patients (Cor = 0.374, *P* = 1.26E-04). In SKCM-metastasis patients, CXCL8 is positively correlated with the infiltration of CD8 + T cells (Cor = 0.135, *P* = 1.34e-02), Macrophage cells (Cor = 0.15, *P* = 4.79e-03), and Neutrophil cells (Cor = 0.303, P = 7.27e-09) and Dendritic cells (Cor = 0.147, P = 6.31e-03). In SKCM-primary patients, down-regulation of CXCL9 enhanced infiltration of CD8 + T cells (COR = 0.573, *P* = 3.65e-10), CD4 + T cells (COR = 0.349, *P* = 3.44e-04), Neutrophil cells (COR = 0.488, *P* = 2.52e-07), and dendritic cells (COR = 0.535, *P* = 7.93E-49). In SKCM-metastasis patients, CXCL9 was positively correlated with infiltration of B cells (COR = 0.201, *P* = 1.73e-04), CD8+ T cells (COR = 0.679, *P* = 8.20e-47), CD4+ T cells (COR = 0.255, *P* = 1.71e-06), Macrophage cells (COR = 0.255, *P* = 1.27e-06), Neutrophil cells (Cor = 0.68, *P* = 6.05E-49) and Dendritic cells (Cor = 0.672, P = 1.48e-46). In SKCM-primary patients, CXCL10 was certainly related to infiltration of CD8 + T cells (COR = 0.584, *P* = 1.40e-10), CD4 + T cells (COR = 0.326, *P* = 8.87e-04), Neutrophil cells (COR = 0.637, *P* = 1.08e-12), and dendritic cells (COR = 0.58, *P* = 2.14e-10). In SKCM-metastasis, CXCL10 was positively correlated with infiltration of B cells (COR = 0.143, *P* = 7.51e-03), CD8+ T cells (COR = 0.613, *P* = 4.29e-36), CD4+ T cells (COR = 0.244, *P* = 4.49e-06), Macrophage cells (COR = 0.226, *P* = 1.94e-05), Neutrophil cells (Cor = 0.697, *P* = 2.32E-52) and Dendritic cells (Cor = 0.614, P = 4.83e-37).

**Table 4:** The Cox proportional hazard model of CXC chemokines and six tumor-infiltrating immune cells in SKCM (TIMER).

	coef	HR	95%CI_l	95% CI_u	*p*-value	sig
B_cell	−2.678	0.069	0.001	3.178	0.171	
CD8_Tcell	−0.137	0.872	0.070	10.780	0.915	
CD4_Tcell	2.554	12.859	0.409	403.809	0.146	
Macrophage	2.543	12.722	0.951	170.184	0.055	*
Neutrophil	−4.938	0.007	0.000	26.239	0.238	
Dendritic	−0.115	0.891	0.105	7.544	0.916	
CXCL1	0.060	1.062	0.946	1.193	0.310	
CXCL2	−0.319	0.727	0.528	1.001	0.051	*
CXCL3	0.104	1.109	0.725	1.697	0.633	
CXCL4	0.108	1.114	0.850	1.461	0.433	
CXCL5	0.093	1.098	0.930	1.296	0.269	
CXCL6	−0.077	0.926	0.693	1.236	0.600	
CXCL7	0.062	1.064	0.837	1.352	0.612	
CXCL8	0.089	1.093	0.975	1.224	0.127	
CXCL9	0.049	1.050	0.865	1.273	0.622	
CXCL10	−0.075	0.928	0.724	1.190	0.556	
CXCL11	−0.071	0.932	0.688	1.261	0.647	
CXCL12	−0.013	0.987	0.864	1.127	0.845	
CXCL13	0.013	1.013	0.880	1.166	0.858	
CXCL14	0.068	1.070	0.993	1.154	0.075	*
CXCL16	−0.145	0.865	0.726	1.031	0.105	
CXCL17	0.175	1.192	1.030	1.378	0.018	*

*P < 0.05.

Similar results were found in CXCL11, CXCL12, CXCL13, CXCL14, and CXCL16. The upregulation of these CXC chemokines enhanced infiltration of B cells, CD8+ T cells, CD4+ T cells, Macrophage cells, Neutrophils cells, and Dendritic cells (**Figures 10H–L**). There is no linear correlation between CXCL17 and various immune cells in SKCM-primary and SKCM-metastasis patients. The confounding factors were corrected by using a Cox proportional hazard model. Macrophage (P = 0.055), CXCL2 (P = 0.051), CXCL14 (P = 0.075), and CXCL17 (P = 0.018) were closed related to the clinical outcome of SKCM patients (b).

### Validation of CXC Chemokines in Clinical Samples

**Figure 11:**
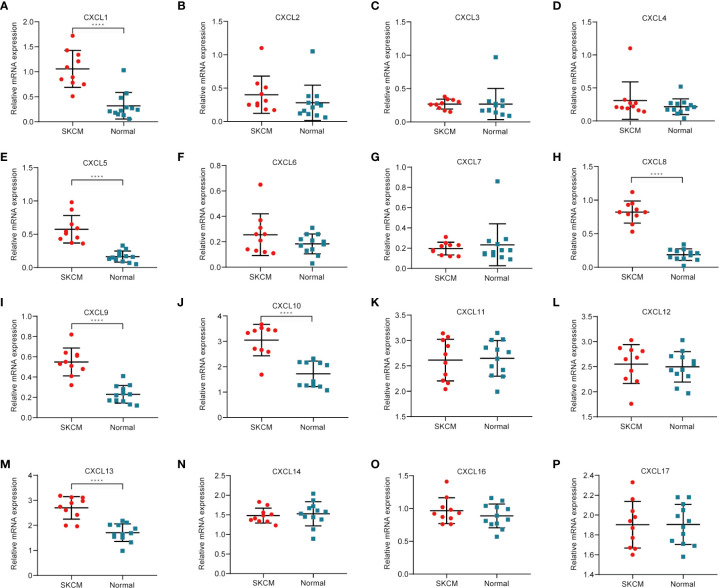
Real-time qPCR validation of CXC chemokines in 10 SKCM and 12 normal skin tissues **(A–P)**. ****P < 0.05, analysis by non-paired tested.

To further determine which genes might play a significant role in the progression of SKCM, real-time PCR was used to detect the expression of CXC chemokines using clinical samples, including CXCL1, CXCL2, CXCL3, CXCL4, CXCL5, CXCL6, CXCL7, CXCL8, CXCL9, CXCL10, CXCL11, CXCL12, CXCL13, CXCL14, CXCL16, and CXCL17 (**Figures 11A–P**). The analysis results showed that CXCL1, CXCL5, CXCL8, CXCL9, CXCL10, and CXCL13 were usually upregulated in SKCM tissues comparing to normal skin tissues, which is consistent with the results of bioinformatics analysis above. There was no significant difference in CXCL2, CXCL3, CXCL4, CXCL6, CXCL7, CXCL11, CXCL12, CXCL14, CXCL16, and CXCL17 between SKCM tissues and normal skin tissues, which is also consistent with the Oncomine analysis results.

## Discussion

Chemokines are tiny protein molecules whose main function is associated with inflammation and immunity (27). Chemokines have been divided into five main subfamilies: CXC, CC, CX3C, XC, and CX (28). CXC chemokines occupy a vital position in the chemokine family. They have been classified into ELR + CXC and ELR − CXC according to the motif with or without Glu leu-ARG (ELR) (29). Chemokine receptors are G-protein-coupled transmembrane receptors that mediate the function of chemokines and contain 7 transmembrane regions (30, 31). CXC chemokines and their receptors are expressed in a variety of cells and play an important role in the generation, differentiation, development regulation, immune response regulation of immune cells, and bone marrow hematopoietic cells (32). They can promote angiogenesis, tumor cell proliferation, survival, and dissemination. They also participate in the process of organ-specific metastasis of malignant tumors and play an important role in the occurrence and development of tumors (33, 34). However, there are few studies on the potential value of CXC’s clinical diagnosis and treatment guidance in SKCM. Therefore, exploring the pathological and molecular mechanisms of CXC chemokines in SKCM is basis for clinical diagnosis and treatment.

To probe CXC chemokines and its relationship with the pathological stage in SKCM patients, mRNA analyses were performed for the 16 CXC chemokines using ONCOMINE and GEPIA. Among the 16 CXC chemokines, six chemokines (CXCL1, CXCL5, CXCL8, CXCL9, CXCL10, and CXCL13) were identified as being expression difference between SKCM patient tissues and normal tissues. ONCOMINE database analysis results showed that the expression levels of these six CXC chemokines in SKCM tissues were significantly higher than that in normal tissues. Further validation by clinical samples, the mRNA level of CXCL1, CXCL5, CXCL8, CXCL9, CXCL10, and CXCL13 was considerably adjusted. One research has pointed out that CXCL1 down-regulation leads to reduced colorectal cell viability, invasion, and proliferation. *In vivo*, knockdown of CXCL1 resulted in the prevention of tumor growth in nude mice (35). CXCL1 promotes tumor growth through VEGF pathway activation and is associated with inferior survival in gastric cancer (36). Therefore, the high expression of CXCL1 might promote melanoma cell viability, invasion, and proliferation. Neutrophils may be the cause of elevated CXC5 expression in primary melanoma (37). The up-regulation of CXCL8 and its receptors CXCR1 and CXCR2 may be one of the reasons for the development of SKCM. CXCL8 promotes tumor angiogenesis by maintaining the proliferation and activity of endothelial cells, and CXCR1 and CXCR2 are the basis of this function (38). CXCL9 promotes the metastasis of melanoma cells through its combination with CXCR3 to enhance the permeability of tumor blood vessels (39). High expression of CXCL10 will inhibit the immune response mediated by T cells, leading to an increase in tumor growth rate (40). The CXCL13:CXCR5 axis is an fundamental regulatory component in the biological process of SKCM (41). Overall, these findings match the results of our data mining. Besides, GEPIA was used to explore whether there is a connection between CXC with the survival rate in SKCM. The increased expression of six CXC chemokines (CXCL4, CXCL9, CXCL10, CXCL11, CXCL12, and CXCL13) was related to tumor progression. The SKCM patients with low expression of them had better overall survival. Previous studies have demonstrated that CXC chemokines may also play an important role in the progression of SKCM (42, 43).

The expression differences of CXC chemokines between primary SKCM and metastatic SKCM were explored by using UALCAN database. The result showed that CXCL1 and CXCL7 were higher in the primary SKCM than in the metastatic SKCM. One research indicates that primary melanoma cells might down-regulate the invasion activity of metastatic melanoma cells through CXCL1 signaling (44). Therefore, we speculated that the high expression of CXCL1 in primary SKCM can inhibit tumor metastasis. So far, no studies on CXCL7 in primary and metastatic SKCM have been found. The studies of other tumors showed that CXCL7 was closely related to tumor metastasis, and the high expression of CXCL7 would cause tumor metastasis. This contradicts the results of our analysis. There are two possible reasons for considering UALCAN database analysis results. The first reason is that the amount of data collected by the UALCAN database is insufficient, so there is a deviation in the analysis results. The second reason is that CXCL7 expression may be higher in primary SKCM than in metastatic SKCM, but there is no specific mechanism to explain. In the future study, samples will be collected for verification and related mechanism exploration.

As suggested by the results of the DAVID analysis, the CXC chemokines and 50 most closely interacting genes were enriched for the terms inflammatory response and immune response in the “biological process” category, the term extracellular space and external side of the plasma membrane in the “cellular component” category, chemokine activity and chemokine receptor binding in the “molecular function” category. The KEGG pathway analysis showed the enrichment of 50 most closely interacting genes for the terms of the chemokine signaling pathway, cytokine-cytokine receptor interaction, and TNF signaling pathway. The enrichment analysis results of Metascape were similar with DAVID. This is following the results reported before, which have shown that CXC chemokines signaling pathways occupy a mayor position in multiple biological activities of tumors (45, 46). A large number of clinical and epidemiological studies have shown that 15–20% of a malignant tumor is caused by infection and inflammation of the controllability, such as inflammatory bowel disease associated with colon cancer, chronic hepatitis B virus infection can lead to liver cancer, *Helicobacter pylori* infection was significantly associated with gastric cancer, EB virus infection can cause nasopharyngeal carcinoma, the human papillomavirus infection can cause cervical cancer or Burkitt lymphoma, *etc.* (47). Chronic inflammation is involved in the pathogenesis, development, invasion, and metastasis of malignant tumors (48). These results indicate that CXC chemokines regulate the progress of SKCM by regulating inflammation and immune responses.

TRRUST database was used to explore the transcription factor targets of CXC chemokines with different expressions. The analysis results showed that the transcription factors which play an important in regulating CXC chemokine are RELA, NFKB1, and SP1. NF-KB pathway was regulated by Phosphorylated RELA which is related to the course of tumor, and inflammation-related diseases (49). RELA has been shown to play a key role in mediating cancer-induced senescence in precancerous lesions (50). NF-kB1 inhibits the occurrence and development of a variety of cancers by reducing the overexpression of the NF-kB signaling pathway (51). Some research has showed that Sp1 is overexpressed in cancer cells and contributes to the formation of cancer (52). Inhibiting the expression level of Sp1 can significantly inhibit the proliferation of human malignant melanoma cells (53). The above results confirmed that RELA, NF-KB, and Sp1 play an important role in CXC chemokines regulation of SKCM. LinkedOmics database was applied to explore the kinase target networks of CXC chemokines in SKCM. The result suggested that LCK, LYN, FYN, MAPK1, MAPK3, and CSNK1D may be targets for differential expression of CXC chemokines. These kinases influence cancer formation and progress by modulating cancer cell migration, invasion, and apoptosis. The results of our observation indicated that these kinases were the important regulator of CXC chemokines in SKCM.

CXC chemokines can mediate the migration and localization of immune cells (54, 55). Timer database was applied to analyze the association between CXC chemokines and immune cells. There is growing confirmation showed that immune cell infiltration may influence cancer development and recurrence and is an important determinant of immunotherapy response and clinical outcome (56, 57). The results showed that CXC chemokines and immune cell infiltration levels were positively correlated in SKCM. Macrophage, CXCL2, CXCL14, and CXCL17 were closely related to the clinical outcome of SKCM patients. These strongly confirmed the positive correlation between CXC chemokines and immune infiltration in SKCM. Thus, it is likely that CXC chemokines not only as being diagnostic biomarkers but also affect the immune condition in SKCM.

There are some limitations in our research. Analysis of transcription can indicate immune condition but not integrated status. Also, vivo and vitro research should be conducted to verify our results. Our findings will offer new understanding to support the program of new immunotherapy, assist doctors select efficacious drugs and prognostic biomarkers for SKCM patients and identify biomarkers to precisely estimate prognosis.

## Data Availability Statement

The original contributions presented in the study are included in the article/**Supplementary Material**. Further inquiries can be directed to the corresponding authors.

## Author Contributions

Conceptualization, XX, WS, and XZZ. Data curation, MP, JP, and CW. Methodology, WS, XZ, and XZZ. Writing, WS, YH, and XZZ. All authors contributed to the article and approved the submitted version.

## Funding

This work was supported by National Nature Science Foundation (No. 81974132, No. 81974134, and No. 81770927). Hunan province also provided financial support in the form of the Human Nature Science Foundation (No. 2018JJ2624).

## Conflict of Interest

The authors declare that the research was conducted in the absence of any commercial or financial relationships that could be construed as a potential conflict of interest.

## Supplementary Material

Supplementary Table 1 The primers used for quantitative RT-PCR.
