# Supplementary material for: CXC Chemokines as Therapeutic Targets and Prognostic Biomarkers in Skin Cutaneous Melanoma Microenvironment
Source: Front Oncol. 2021 Mar 9;11:619003. doi: 10.3389/fonc.2021.619003 (PMC7985846; doi:10.3389/fonc.2021.619003)
Supplement: Supplementary Table 1 — The primers used for quantitative RT-PCR. [file Table_1.docx]

Supplementary Table 1. The primers used for quantitative RT-PCR.

| **Gene** | **Forward (5’＞3’)** | **Reverse (5’＞3’)** |
| --- | --- | --- |
| CXCL1 | GCGCCCAAACCGAAGTCATA | ATGGGGGATGCAGGATTGAG |
| CXCL2 | AACCGAAGTCATAGCCACAC | CTTCTGGTCAGTTGGATTTGC |
| CXCL3 | AAGTGTGAATGTAAGGTCCCC | GTGCTCCCCTTGTTCAGTATC |
| CXCL4 | TCCTGCCACTTGTGGTCGCCT | CCTTGATCACCTCCAGGCTGG |
| CXCL5 | CAGACCACGCAAGGAGTTCATC | TTCCTTCCCGTTCTTCAGGGAG |
| CXCL6 | GTCCTTCGGGCTCCTTGTGC | GGGGCTTCCGGGTCCAGA |
| CXCL7 | TGCTCTGGCTTCCTCCACCAAA | ACACATGCAGCGGAGTTCAGCA |
| CXCL8 | GAGAGTGATTGAGAGTGGACCAC | CACAACCCTCTGCACCCAGTTT |
| CXCL9 | CTGTTCCTGCATCAGCACCAAC | TGAACTCCATTCTTCAGTGTAGCA |
| CXCL10 | TGCCATTCTGATTTGCTGCC | TGCAGGTACAGCGTACAGTT |
| CXCL11 | AAGGACAACGATGCCTAAATCCC | CAGATGCCCTTTTCCAGGACTTC |
| CXCL12 | CTCAACACTCCAAACTGTGCCC | CTCCAGGTACTCCTGAATCCAC |
| CXCL13 | TATCCCTAGACGCTTCATTGATCG | CCATTCAGCTTGAGGGTCCACA |
| CXCL14 | AGATCCGCTACAGCGACGTGAA | GCAGTGCTCCTGACCTCGGTA |
| CXCL16 | CCTATGTGCTGTGCAAGAGGAG | CTGGGCAACATAGAGTCCGTCT |
| CXCL17 | ACAGTGTCTGGGCTGCCAAAGA | GGCTCTGGAATGCTTGTTTGGC |
| GAPDH | CCAGAACATCATCCCTGCCT | CCTGCTTCACCACCTTCTTG |
